# The Role of Echocardiography in the Optimization of Cardiac Resynchronization Therapy: Current Evidence and Future Perspectives

**DOI:** 10.2174/1874192401711010133

**Published:** 2017-12-19

**Authors:** Michael Spartalis, Eleni Tzatzaki, Eleftherios Spartalis, Christos Damaskos, Antonios Athanasiou, Efthimios Livanis, Vassilis Voudris

**Affiliations:** 1 Division of Cardiology, Onassis Cardiac Surgery Center, Athens, Greece; 2 Laboratory of Experimental Surgery and Surgical Research, University of Athens, Medical School, Athens, Greece; 3 Department of Surgery, Mercy University Hospital, Cork, Ireland

**Keywords:** Echocardiography, Resynchronization, CRT, Dyssynchrony, Optimization

## Abstract

**Background:**

Cardiac resynchronization therapy (CRT) has become a mainstay in the management of heart failure. Up to one-third of patients who received resynchronization devices do not experience the full benefits of CRT. The clinical factors influencing the likelihood to respond to the therapy are wide QRS complex, left bundle branch block, female gender, non-ischaemic cardiomyopathy (highest responders), male gender, ischaemic cardiomyopathy (moderate responders) and narrow QRS complex, non-left bundle branch block (lowest, non-responders).

**Objective:**

This review provides a conceptual description of the role of echocardiography in the optimization of CRT.

**Method:**

A literature survey was performed using PubMed database search to gather information regarding CRT and echocardiography.

**Results:**

A total of 70 studies met selection criteria for inclusion in the review. Echocardiography helps in the initial selection of the patients with dyssynchrony, which will benefit the most from optimal biventricular pacing and provides a guide to left ventricular (LV) lead placement during implantation. Different echocardiographic parameters have shown promise and can offer the possibility of patient selection, response prediction, lead placement optimization strategies and optimization of device configurations.

**Conclusion:**

LV ejection fraction along with specific electrocardiographic criteria remains the cornerstone of CRT patient selection. Echocardiography is a non-invasive, cost-effective, highly reproducible method with certain limitations and accuracy that is affected by measurement errors. Echocardiography can assist with the identification of the appropriate electromechanical substrate of CRT response and LV lead placement. The targeted approach can improve the haemodynamic response, as also the patient-specific parameters estimation.

## 1 INTRODUCTION

Cardiac resynchronization therapy (CRT) is an established medical therapy for patients with advanced heart failure characterized by left ventricular (LV) dysfunction with ejection fraction ≤35%, LV dyssynchrony with QRS duration ≥120 ms, intraventricular conduction delay and New York Heart Association (NYHA) class II-IV, despite optimal medical therapy.

CRT reduces morbidity and mortality in chronic heart failure [1]. The responses for CRT have been predominantly assessed by LV reverse remodeling, which is mostly defined as a ≥10-15% reduction of LV end systolic volume post‐implantation. Based on the current guidelines, the rate of CRT responders is from 60-70% [2]. The suitability of echocardiographic parameters to improve the response rate to CRT has been controversial. Speckle tracking echocardiography (STE) has been reported as a useful imaging method to detect CRT responders [3, 4]. Atrioventricular (AV) and interventricular (VV) delay optimization can augment ventricular function in CRT and is usually performed utilizing echocardiography [5]. The conflicting evidence can be attributed to a combination of factors including poor precision, lack of experience, short follow-up time, late responders, or failure to distinguish non-responders. CRT benefit can be maximized through optimization of pacing site location or device pacing parameters [6].

## 2 MATERIALS AND METHODS

The MEDLINE/PubMed database was searched for publications with the medical subject heading “echocardiography” and keywords “resynchronization” or ‘’resynchronisation” or “CRT”, “echo” and “resynchronization” or “CRT” and “echocardiographic” and “resynchronization”. Additional records were identified through scanning bibliographies of relevant articles. Our selection criteria were the English language, the cardiovascular relevance (publications irrelevant to echocardiography and cardiac resynchronization therapy, were excluded), a time frame of the last 5 years (2012-2017), and the availability of full text articles. We included 70 articles. Our aim was to review the effect of echocardiography on optimization of CRT before, during and after implantation. A comprehensive Preferred Reporting Items for Systematic Reviews and Meta-Analyses (PRISMA) flow diagram with exclusion criteria is reported in Fig. (**1**).

## 3 RESULTS

### 3.1 Current Evidence for Echocardiographic CRT Optimization

The 2013 ACCF/HRS/AHA/ASE/HFSA/SCAI/SCCT/SCMR guidelines on cardiac pacing suggest posterolateral LV lead position, the target of latest activated area and avoidance of apical position [2]. A shortest AV delay without truncation of the A-wave (Ritter's method) or change in LV systolic function is suggested, and a residual LV dyssynchrony and the largest stroke volume by Echo Doppler is recommended as CRT optimization about VV delay [2]. The 2016 European Society of Cardiology guidelines for the diagnosis and treatment of acute and chronic heart failure suggest that echocardiography may be considered only for patients who have had a disappointing response to CRT [7-12].

### 3.2 The Role of Echocardiography in Predicting Response

Although the electrocardiogram (ECG) has provided strong predictors of CRT response like QRS width and left bundle branch block (LBBB), it is less sensitive than echocardiography in detecting lesser degrees of mechanical dyssynchrony. The multi-centre Predictors of Response to CRT (PROSPECT) trial studied 12 echocardiographic parameters of dyssynchrony and found that although several parameters were different between CRT responders and non-responders, they showed only modest sensitivity and specificity. The investigators concluded that the accuracy of echocardiography is affected by measurement errors and no single parameter could reliably improve patient selection for CRT [8, 13].

### 3.3 Optimization Before Implantation

Ventricular dyssynchrony is a primary electrical disease induced by deficits in infrahisian conduction that leads to mechanically inefficient cardiac pump function. Ventricular dyssynchrony typically manifests in the form of LBBB and affects roughly one-third of patients with symptomatic heart failure [14]. The consequences of such include decreased ejection fraction, decreased exercise tolerance and increased mortality [14]. In patients with LBBB, there is a delay in the activation of the left lateral wall. The outcome is that early in systole, unopposed ventricular septal contraction causes stretch of the still quiescent lateral wall. In late systole, there is a delay in lateral wall contraction that occurs against an already pressurized blood pool, leading to elevated wall stress, poor mechanical function, and even abnormal myocardial expression of mediators of the stress response, calcium handling and myocyte coupling [14]. Ischaemic patients, by definition, have fibrosis and scarring of the ventricular myocardium. Non-ischaemic patients, though, also have been shown to have significant burdens of ventricular scarring. Global scar burden predicts a worse outcome than the decreased LVEF alone, showing that the electrical abnormalities in scarred myocardium pose as an additional burden [14]. Echocardiography and MRI help localize regions of the scar so that leads can be placed over healthy myocardium [15]. In this frame of analysis, the utilization of at least 1 of the 2 imaging modalities in preoperative scheduling can play a major role, as neither the surface ECG nor the intraoperative threshold calculations are sufficiently accurate at localizing myocardial scar and avoiding the problems that follow pacing in adjacent segments [14].

Correcting mechanical dyssynchrony is suggested as the predominant mechanism of response. Achieving optimum left ventricular lead position, at the site of maximal mechanical dyssynchrony but away from the transmural scar, is identified as one of the main determinants of both symptomatic and prognostic benefit [16, 17]. Speckle tracking echocardiography (STE) is a clinically reproducible method of assessing LV dyssynchrony and offers prospective lead targeting, integrating pathophysiological determinants of CRT response [16, 18, 19]. 3D echocardiography STE is more useful in predicting response in non-ischaemic patients than ischaemic patients [20].

STE has the benefit of relatively less angle dependence and the capability to calculate strain in all 3 planes of cardiac motion. STE also can provide information on patterns of myocardial activation, thus allowing the identification of optimal LV lead positioning pre-CRT implantation [8, 10]. Saba *et al.* in the Speckle Tracking Assisted Resynchronization Therapy for Electrode Region (STARTER) trial established radial strain by STE as a reliable echo-guided LV lead placement method [21]. The study enrolled 187 NYHA II to IV patients randomized to the echo-guided group and the routine group. The echo-guided group had a success rate of 85% and an improvement in event-free survival and the routine group a fortuitous success rate of 66% [21, 22]. Mechanical dyssynchrony assessment by STE has an incremental value to predict CRT responders [23]. STE contributes to decision making for CRT indications especially if non-responders are to be avoided in the clinical setting [3].

Adelstein *et al.* in the STARTER trial compared patients treated with CRT-D and echo-guided LV lead placement in the region of the latest mechanical activation with a group of conventional implantation [24]. The echo-guided LV lead placement strategy was superior and improved the patient survival rate free from defibrillator therapy [24]. Abu Daya *et al.* also confirmed the use of echo-guided LV placement in ischaemic and non-ischaemic cardiomyopathy patients. Abu Daya *et al.* investigated patients from STARTER trial and concluded that the strategy of echo-guided LV lead placement improved the result of CRT-D therapy-free survival primarily in ischaemic patients and the outcome of HF hospitalization-free survival in both ischaemic and non-ischaemic patients [25].

Bakos *et al.* even used a combined bullseye plot from speckle tracking radial strain echocardiography, cardiac CT scan, and MRI scan, in order to choose the optimal electrode position [26]. Brunet-Bernard *et al.* used a combined score with clinical (age >70 years, non-ischaemic origin), electrocardiographic (LBBB), and echocardiographic characteristics **(**left ventricular end-diastolic diameter <40 mm/m, septal flash) as a response predictor. The score has a 71% probability rate and is an easy tool for the clinician [27]. Park *et al.* utilized an echocardiographic score with multiple parameters (LV end-diastolic dimension index <3.1 cm/m^2^, global longitudinal strain of LV <-7%, LA area <26 cm^2^, right ventricular end-diastolic area index <10.0 cm^2^/m^2^, RA area <20 cm^2^, and RV fractional area change ≥35%). 334 patients were enrolled in the study, and the prediction of LV reverse remodeling had a specificity of 79% and a sensitivity of 84% [28].

On the other hand, another study by Badran *et al.* showed that 3D echocardiography guided LV lead placement added no clinical benefit compared with standard techniques [29].

Not all LBBBs by ECG reflect a true LV activation delay. Studies of LV endocardial mapping have reported that up to one-third of patients with LBBB are misdiagnosed. True LBBB activation causes a unique contraction pattern of opposing wall motion with apical rocking motion [30]. Risum *et al.* showed that among patients with LBBB by ECG, those with a typical LBBB contraction pattern developed a significant improvement in response to CRT compared with those without typical LBBB contraction. Patients without a typical LBBB contraction pattern had a 3-fold increased risk of adverse outcome following implantation [30]. The assessment of LBBB-specific contraction by 2D strain echocardiography improved risk prediction beyond ECG (QRS duration and morphology) and aetiology [30].

In a single-centre study by Seo *et al.*, 81 patients undergoing CRT, of whom 50 had LBBB, were enrolled. The U-shaped propagation pattern from the mid-septum to the lateral or posterior wall through the apex on 3D STE was significantly associated with a favourable CRT response [31].

In an analysis of 313 heart failure patients (non-ischaemic, LBBB) who underwent implantation of a biventricular device, the study showed markedly lower longitudinal strain, suggesting that myocardial dysfunction burden might be the predictor of reverse remodeling in these patients [32, 33].

In another study of 200 CRT patients, the echocardiography examination included the evaluation of mechanical abnormalities amenable to correction with CRT. These were septal flash (inward/outward motion) of the septum occurring during the isovolumetric contraction period (within the QRS width). Also, abnormalities in LV filling in the absence of septal flash, including either fused E and A waves with diastolic mitral regurgitation (extended AV delay) or a truncated A-wave (short left AV delay) and an exaggerated right–left interaction without a concomitant septal flash or an abnormal filling [34]. The patients were followed-up 12 months after device implantation at the outpatient clinic. Echocardiographic response to CRT was defined as a reduction of left ventricular end-systolic volume (LVESV) ≥15% at follow-up in the absence of death or heart transplantation [34] and a reduction of LVESV ≥10% [35]. The presence of these mechanical abnormalities proved to be an independent predictor of response and midterm mortality, along with creatinine level and left ventricular end-systolic diameter (LVESD). The main advantage is that it would prevent the implantation of a CRT device in patients without mechanical abnormalities and, thus, a strong possibility of nonresponse (94.2%) [34].

In a study by van't Sant *et al.* of 205 CRT patients, LVESV found to be an excellent surrogate marker of CRT response concerning long-term outcome for non-ischaemic patients but a poor surrogate marker of CRT response in ischaemic patients [36].

Yu *et al.* examined 227 patients with echocardiography using left ventricular end-diastolic volume (LVEDV). To some extent, LVEDV reflects diastolic function which is crucial for cardiac filling volume, while LVESV determines pumping volume. Severe diastolic dysfunction is hardly reversed by CRT so that LVEDV plays a major role in predicting CRT non-response. LVEDV >255 mL proved to be a valuable predictor of CRT non-response [37].

A Chinese study by Zhao *et al.* used systolic dyssynchrony index (SDI), a quantification parameter by 3D echocardiography to assess the effects of CRT. SDI was well correlated with the increase in LVEF, proving to be a useful predictor of CRT response [23, 38, 39].

There is limited evidence about the role of echocardiography in children. There is 1 study examining 19 children with structural heart disease and CRT. Children have limited capacity for cooperation. According to the authors, echocardiography optimization of synchrony was not superior to ECG optimization. ECG optimization required less time and cost [40, 41].

Wita *et al.* investigated the reverse LV remodeling of 57 CRT patients before implantation with Tissue Doppler imaging (TDI) echocardiography. The authors concluded that the exercise intraventricular dyssynchrony assessed by dobutamine stress echo (DSE) is a strong independent predictor of CRT response [42].

In this frame of analysis, in a study of 127 patients with heart failure, low-dose dobutamine response was determined by echocardiography before CRT. Results reported by Płońska-Gościniak *et al.* , suggesting a significant relationship between LV contractile reserve at DSE and long-term all-cause mortality following CRT implantation. Septal flash and interventricular dyssynchrony were also predictive of the response to resynchronization, but the presence of myocardial viability was not [43].

Murin *et al.* studied 52 symptomatic patients with heart failure before CRT implantation. Myocardial contractile reserve assessed by high-dose DSE proved to be a major factor in identifying responders to CRT, as responders showed a greater increase in EF compared with non-responders [44]. Mitro *et al.* assessed regional contractile reserve by strain rate echocardiography and reached the conclusion that responders showed a significant increase in regional deformation compared with non-responders [45]. All of the above suggest that contractile reserve is a strong predictor of response to CRT [46].

Finally, Holmqvist *et al.* examined 892 patients from the multi-centre automatic defibrillator implantation trial with cardiac resynchronization therapy (MADIT-CRT). An atypical P-wave morphology was associated with reverse remodeling response at 1 year [47].

### 3.4 Optimization During Implantation

Moubarak *et al.* investigated if CRT could be optimized during the implantation by choosing the location and number of pacing sites using echocardiography guidance. The objective of the optimization process was to enhance LV efficiency and to reduce the left pre-ejection interval (time interval between the beginning of QRS and the onset of LV ejection) as much as possible compared with a standard configuration. Ninety-one patients were studied and showed that intraoperative echo-guided placement of RV lead(s) during CRT implantation is feasible and acutely enhances LV synchrony compared with standard biventricular stimulation [48].

Zhang *et al.* utilized transoesophageal echocardiography in 14 patients where LV placement was not feasible. The study investigated the use of transoesophageal echocardiography in LV lead epicardial placement with a surgical approach. TDI was used to select the optimum LV lead placement. Minimally invasive surgical placement of the LV epicardial lead is feasible, safe, and efficient. TDI guidance contributes to the epicardial lead placement to the ideal target location [49].

### 3.5 Optimization after Implantation

Echocardiography can be used to optimize atrioventricular and interventricular delays (AV/VV delay). AV-delay optimization influences ventricular filling and may cause fusion with intrinsic conduction, thereby also affect intraventricular and interventricular interaction. VV-delay optimization also influences intraventricular and interventricular dynamics, leading to more homogeneous LV contraction [11]. On the other hand, a meta-analysis with a total of 4356 patients treated with CRT, showed no benefit from AV and VV optimization compared with empiric device programming [50].

Burns *et al.* examined 294 patients after CRT implantation; 120 patients were non-responders after 1 year. Burns *et al.* investigated a delayed response using echocardiography; 53 patients (43%) experienced a delayed response after 3 years [51].

Rocha *et al.* in a prospective study observed 116 patients after CRT. The study compared echocardiographic evaluation pre-implantation and 6 to 12 months after implantation. The parameters of EF <30%, severe diastolic dysfunction and severe mitral regurgitation were independently related to increased cardiac mortality [52]. Another study tried to correlate right ventricular function with response prediction but failed to provide any results. There was no significant value in differences in baseline right ventricular function between responders and non-responders [53]. Abu Sham'a *et al.* correlated worsened tricuspid regurgitation after CRT with poor clinical response [54].

A prospective observational trial of CRT (PROSPECT) examined 426 patients. The result was a correlation between E/A ratio and LA area with an adverse outcome in CRT patients [55].

In addition, there are other optimization methods which could be used in combination with echocardiography. Noda *et al.* reported that PEP/left ventricular ejection time (LVET) calculations by impedance cardiography (ICG) and echocardiography were positively correlated [56]. Thus, ICG could be useful in combination with echocardiography for CRT optimization.

In a prospective, multicentre, randomized, double-blind controlled clinical trial (the Frequent Optimization Study Using the QuickOpt Method) investigated CRT optimization using an intracardiac electrogram-based approach (Quickopt algorithm provides a calculation of AV and VV delays by intracardiac electrograms) compared with current standard echo-guided approach [57]. The trial failed to present any substantial difference in CRT response between the 2 methods [58].

The Quicksept study showed that aortic velocity time integral (aVTI) values at the optimized AV and VV intervals as determined by echocardiography and by the QuickOpt algorithm were quite well correlated and that this correlation was maintained in long-term follow-up [5, 59]. AV and VV delay optimization data were collected in 13 centres using both echocardiographic and QuickOpt guidance in CRT-D implanted patients provided with this algorithm [5].

The SmartDelay determined AV optimization: a comparison of AV optimization methods used in cardiac resynchronization therapy (SMART-AV) trial prospectively randomized 1014 patients to a fixed empirical AV delay (120 msec), echo-optimized AV delay, or AV delay optimized with SmartDelay, an intracardiac electrogram-based algorithm [60]. The trial showed no difference in 6-month follow-up in LVESV, NYHA status, quality of life or 6 min walk test [60].

To add to the confusion, Singh *et al.* compared clinical response between adaptive cardiac resynchronization therapy (aCRT), a novel algorithm for CRT pacing with AV delay optimization with echocardiography. Adaptive CRT provides automatic ambulatory selection between synchronized left ventricular (LV) or bi-ventricular (BiV) pacing, and optimization of atrioventricular (AV) and interventricular (VV) delays based on periodic measurement of intrinsic conduction. The authors concluded that aCRT provided additional clinical benefit compared with CRT with only AV delay optimization post-implantation [6].

Starling et al analysed data from the Adaptive CRT trial and concluded that the use of the aCRT algorithm was associated with a significant reduction in the probability of a 30-day readmission after both heart failure and all-cause hospitalizations [61].

Whinnet *et al.* applied non-invasive blood pressure monitoring, by continuous finger photoplethysmography, to directly detect the haemodynamic response during adjustment of the AV delay of CRT, at different heart rates [62]. The blood pressure monitoring demonstrated that even small changes in AV delay from its haemodynamic peak value had a significant effect on blood pressure [62].

Pappone *et al.* compared 44 patients randomized to a multi pacing point (MPP) group and a routine biventricular group. Both groups were evaluated with echocardiography pre and after implantation. MPP resulted in an improved rate of response to CRT [63]. Another study confirmed similar results. In this study, Calo *et al.* investigated 11 patients with CRT, which received a quadripolar LV lead. MPP resulted in significant reduction in LVEDV and LVESV. MPP with optimal LV pacing configuration by echocardiography is associated with a significant improvement in NYHA class and EF after 6 months [64]. Siciliano *et al.* compared MPP with BiV using 3D echocardiography and echocardiographic particle imaging velocimetry (Echo-PIV). The results regarding LVEF and cardiac output were similar to both. There was an improvement in global longitudinal and circumferential strain, but without statistical significance with MPP [65, 66].

Table (**1**). summarizes the echocardiographic parameters for patient selection and response prediction.

## 4 DISCUSSION

Currently, no echo technique is accepted or guideline-endorsed for the identification of CRT responders [2]. This is also reflected in the 2016 ESC guidelines for the diagnosis and treatment of acute and chronic heart failure, which do not recommend using the presence of echocardiographic dyssynchrony as selection criteria for CRT [8].

The 2013 appropriate use criteria for implantable cardioverter-defibrillators and CRT also do not recommend a routine AV and VV optimization, which should be restricted to non-responders and patients with ischaemic heart disease [2].

2D and 3D speckle-tracking echocardiography can be used in the assessment of dyssynchrony, in conjunction with echocardiographic parameters that may hold prediction potential for the CRT response. Additionally, STE can contribute to optimal LV lead placement guidance pre-implantation [8].

Speckle tracking echocardiography is a technique that needs to be incorporated into routine practice to guide the implant strategy in a more personalized patient-specific approach [16].

Optimization with Doppler and 3D echocardiography is superior to ECG optimization [67].

The existence or lack of an electrical activation delay is a significant reason why some patients are CRT responders, and others are not. The establishment of the underlying electrical substrate for CRT by assessment of the mechanical dyssynchrony using strain echocardiography may be clinically useful [30]. The importance of the contraction pattern is independent of QRS duration, therefore can be particularly beneficial for selection of patients with QRS duration between 120 and 150 ms and LBBB, in which the role for CRT is still debated [30].

Mechanical dyssynchrony is caused not only by electrical dyssynchrony but from abnormalities in regional contractility of the LV and loading conditions also. New parameters of electromechanical dyssynchrony based on this approach and can be obtained by echocardiography are septal flash, LBBB-typical pattern by longitudinal strain, apical rocking, septal strain patterns, and systolic stretch index [68]. These methods could be used in screening patients whom the benefit of CRT remains uncertain like patients with intermediate QRS width (120-150 ms) [68].

Multimodality pre-implantation evaluation and leadless implantable pacemakers may free electrophysiologists from the constraints of the coronary sinus, in order to make nonresponse to CRT increasingly rare [14].

Multipoint pacing (MPP) superiority over biventricular pacing (BiV) needs larger echocardiography driven studies to confirm the hypothesis [65].

Patients with LVEF >35 % (especially patients with LBBB) are an interesting group for further research. These individuals might benefit from CRT [11].

Echocardiographic follow-up period in non-responders after CRT implantation needs to be reassessed. Nearly half of the Burns *et al.* study non-responders developed delayed response after 3 years [51]. The follow-up period was 6 to 12 months.

An ongoing trial by Donazzan *et al.* (CARTEDO trial) is investigating AV and VV optimization 12 months after implantation with a primary goal to evaluate the benefit of late echo-guided adjustments [69].

Versteeg *et al.* in a 54 patients study found a significant discrepancy between echocardiographic and patient-reported health status response to CRT. In the 54 patients with divergent responses, 25 of the patients (22.9%) had an echocardiographic response, but no clinical status response and 29 of them (26.6%) had a clinical status response but no echocardiographic response [70].

Large prospective studies should be designed to evaluate the role of echocardiography in patient selection, response prediction, and CRT optimization.

## CONCLUSION

Echocardiography has a pivotal role in CRT, underlined by the vast field of application, defining cardiac function and particularly left ventricular (LV) function and response due to desired reverse electro-mechanical remodeling. LVEF along with certain electrocardiographic criteria remains the cornerstone of CRT patient selection.

The range from Doppler imaging to deformation imaging to 3D echocardiography has shown promise, and emerging evidence will shed light if they can indeed offer the possibility of patient selection, response prediction, lead placement optimization strategies and optimization of device configurations. Additionally, alternative CRT optimization methods have failed to provide consistent improved clinical outcomes and CRT response.

The results of our present analysis lead to the conclusion that echocardiography is a non-invasive, cost-effective, highly reproducible method with known limitations and accuracy that is affected by measurement errors. Echocardiography can assist with the identification of the appropriate electromechanical substrate of CRT response and LV lead placement at the site of the latest mechanical activation. The targeted approach can improve the haemodynamic response and patient-specific parameters estimation.

It is also evident that studies on the role of echocardiography on optimization of CRT frequently lead to contradictory results. It is not known whether these differences should be attributed to study design, lead misplacement or to the difficulty to understand the exact mechanism of dyssynchrony, myocardial scar, irreversible advanced heart failure and AV/VV optimization.

We believe that the role of echocardiography in the optimization of CRT deserves further experimental investigation and large-scale prospective randomized clinical trials.

## Figures and Tables

**Fig. (1):**
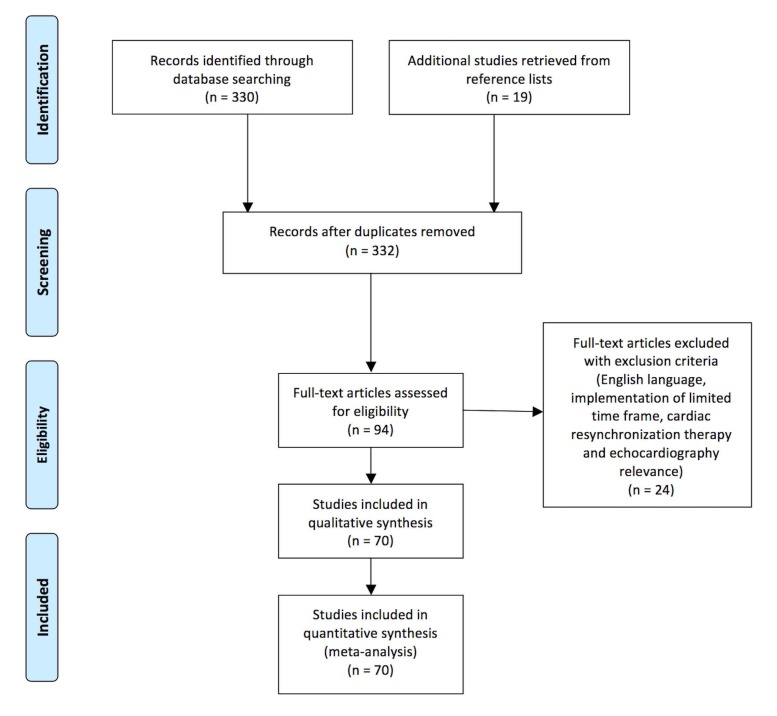
PRISMA flow diagram with exclusion criteria for the selection of sources for the purpose of the review.

**Table 1:** Summary of the echocardiographic parameters for patient selection and response prediction. RV: right ventricle, IVMD: interventricular mechanical delay, LV: left ventricle, LVPEP: left ventricular pre-ejection period, RVPEP: right ventricular pre-ejection period, PW: pulsed wave, LVOT: left ventricular outflow tract, RVOT: right ventricular outflow tract, TDI: tissue doppler imaging, PLAX: parasternal long-axis view.

Parameter	Description	View	Cutt-off	Advantages	Disadvantages
**Apical rocking**	Visual assessment of apical transverse motion	Apical 4-chamber view	Yes/No	Highly reproducible method, high specificity for response prediction	Affected by RV function
**Septal flash**	Visual assessment of short inward septal motion during beginning of systole	Apical 4-chamber view	Yes/No	Highly reproducible method, high specificity for response prediction	Translation of continuous process to on/off phenomenon, observer differences
**IVMD**	Interventricular mechanical delay, difference in onset of outflow of LV (LVPEP) and RV (RVPEP)	PW Doppler of LVOT and RVOT	40 msec	Highly reproducible method	Affected by both LV and RV function
**Septal strain patterns**	Strain pattern of the septum during systole	Apical 4-chamber view	3 types (1,2 responder/ 3 non-responder)	Prediction of volumetric response and outcome	Technically demanding
**SD-TTP**	Standard deviation of time to peak shortening (strain) or velocity (TDI) of all myocardial segments	Apical 4-chamber view, 2-chamber view, PLAX view	> 32 msec	Offline analysis	Requires high quality image, confounded by passive motion tethering
**SL delay**	Difference of time to peak velocity of septal and lateral view	Apical 4-chamber view	> 65 msec	Prediction of volumetric response and outcome	Confounded by passive motion tethering
**SDI**	Time to minimal systolic volume of 16 segments	3D	9.8%	High value for response prediction	Limited spatial and temporal resolution
**SRSsept** **(Systolic rebound stretch of the septum)**	All positive deflections after initial shortening of the septum during systole	Apical 4-chamber view	4.7%	Prediction of volumetric response and outcome	Technically demanding, observer differences

## LIST OF ABBREVIATIONS

**aCRT**: Adaptive cardiac resynchronization therapy
**AV**: Atrioventricular delay
aVTI: Aortic velocity time integral
BiV: Biventricular pacing
CRT: Cardiac resynchronization therapy
CRT-D: Cardiac resynchronization therapy defibrillator
**CT**: Computed tomography
**DI**: Dyssynchrony index
DSE: Dobutamine stress echocardiography
**dp/dt**: Delta pressure/delta time, rate of rise of left ventricular pressure
ECG: Electrocardiogram
EF: Ejection fraction
ESC: European society of cardiology
**HF**: Heart failure
IVMD: Interventricular mechanical delay
LA: Left atrium
**LBBB**: Left bundle branch block
**LPEI**: Left pre-ejection interval
**LVEDV**: Left ventricular end-diastolic volume
**LVEF**: Left ventricular ejection fraction
**LVESV**: Left ventricular end-systolic volume
**LVOT**: Left ventricular outflow tract
LV: Left ventricle
MPP: Multipoint pacing
**MRI**: Magnetic resonance imaging
**NYHA**: New York heart association
PEP: pre-ejection period
PIV: Particle imaging velocimetry
PW: Pulsed-wave Doppler
RV: Right ventricle
RVOT: Right ventricular outflow tract
SDI: Systolic dyssynchrony index
Sm: Mitral annular peak systolic velocity
SPWMD: Septal posterior wall motion delay
STE: Speckle tracking echocardiography
TDI: Tissue Doppler imaging
Ts: Time to peak systolic velocities
Ts-SD: Time to peak systolic velocities standard deviation
VV: Interventricular delay
